# Natural Occurrence of Ochratoxin A in Musts, Wines and Grape Vine Fruits from Grapes Harvested in Argentina

**DOI:** 10.3390/toxins2081984

**Published:** 2010-08-03

**Authors:** María Lorena Ponsone, María Laura Chiotta, Mariana Combina, Adriana Torres, Patricia Knass, Ana Dalcero, Sofía Chulze

**Affiliations:** 1 Departamento de Microbiología e Inmunología, Facultad de Ciencias Exactas, Físico, Químicas y Naturales, Universidad Nacional de Río Cuarto, Ruta Nacional N 36 Km. 601, (5800) Río Cuarto, Córdoba, Argentina; Email: lponsone@exa.unrc.edu.ar (M.L.P.); mchiotta@exa.unrc.edu.ar (M.L.C.); atorres@exa.unrc.edu.ar (A.T.); adalcero@exa.unrc.edu.ar (A.D.); 2 Instituto Nacional de Tecnología Agropecuaria (INTA), Luján de Cuyo, Mendoza, Argentina; Email: mcombina@mendoza.inta.gov.ar; 3 Members of the Research Career of CONICET, Argentina; 4 Facultad de Ciencias Exactas, Químicas y Naturales, Universidad Nacional de Misiones, Posadas, Misiones, Argentina; Email: patrisk@arnet.com.ar

**Keywords:** must, wines, dried vine fruits, Ochratoxin A, Argentina, immunoaffinity columns

## Abstract

In this study, ochratoxin A (OTA) occurrence in Argentinean musts, wines and dried vine fruits was evaluated, alongside with the performance of OchraStar^TM^ columns for OTA extraction. In all the three matrices analyzed, the OchraStar^TM^ columns showed good performance. The analysis of natural occurrence of OTA in the red must and the red wine samples showed low incidence with low levels of mean OTA contamination (0.12 ng/mL and 0.37 ng/mL, respectively), while 60% of the dried vine fruit samples were contaminated with OTA, in levels ranging from 0.26 to 20.28 ng/g.

## 1. Introduction

Ochratoxin A (OTA) is a widely distributed mycotoxin produced by several species of *Aspergillus* and *Penicillium* genera under diverse environmental conditions. OTA has been shown to be nephrotoxic, hepatotoxic, teratogenic, and immunotoxic to several animal species and to cause kidney and liver tumors in mice and rats [1,2]. The IARC (International Agency for Research on Cancer) has classified OTA as a possible carcinogen to humans (Group 2B) [1]. This toxin occurs in various plant products such as cereals [3], beans, groundnuts, spices [4], dried fruits [5,6,7,8,9,10,11,12], coffee [13], milk and beer [14,15], and in grape by-products such as wine, grape juice, and dried vine fruits [16,17,18]. Grapes are products which have different destinations. One of the most important grape by-products in the national and international market is wine production, this is a product widely consumed by adult individuals, and it may represent, after cereals, a major source of daily OTA intake for this population. Provisional estimates of Codex Alimentarius Commission, based on limited data, suggest that 15% of the total intake of this toxin in Europe is due to wine [19]. It has been shown that early veraison and harvesting time are the critical period for OTA accumulation in grape berries [20,21] and its accumulation can be related to various factors such as geographical area, meteorological conditions, mycoflora composition, grape management, wounds in berries caused by insects, cultivar susceptibility and wine-making techniques [19,22]. 

Ochratoxin A content reduction is remarkable during the vinification process [23,24]. Therefore, using musts with low OTA levels will be possible to produce wines with toxin levels below the limits set by the European Commission (EC) 2 μg/kg [19]. 

Another destination for grapes is their dehydration to elaborate dried vine fruits, which are the products made with healthy dried grapes of *Vitis vinifera L*. They are consumed as dried fruits and are also ingredients in cereal-based foods, such as cereal bars, biscuits, cookies, puddings and breads, among other foods, many of which are consumed by the child population in spite of the risk involved [25]. Argentina occupies the 10th place in global production and export, with the United States and Brazil being the major international buyers. Ninety five percent of the national production of dried grapes is concentrated in San Juan province and a high percentage is exported to international markets [26].

The method used to reduce the water activity (*a*_W_) of the grapes consists mainly of sun-drying in the open-air and the quality is thus dependent on consistent weather conditions [27]. This substrate is kept exposed to high temperatures and sun irradiation for a prolonged period of time, this fact determines the consequent contamination with different fungal species. When intermittent sunshine and rain episodes occur, drying can be slowed down and this can lead to colonization by *Aspergillus* section *Nigri* species, such as *A. carbonarius* [28], and the risk of OTA contamination.

Several high-performance liquid chromatography (HPLC) methods with fluorometric detection (FLD) have been reported for the determination of OTA in wine or dried vine fruit, and two of them were successfully validated through collaborative studies, namely, for wine and beer [29] and for dried vine fruits [30]. The method of Visconti *et al.* [29] has been adopted as the official method by the Association of Official Analitycal Chemists International (AOAC) [29], the European Committee for Standardization (CEN) [31], and the Organisation Internationale de la Vigne et du Vin [32]. Much less effort has been devoted to the analysis of OTA in grape berries and musts. A reliable method for the determination of this toxin in grape berries is necessary for quality control and research purposes aiming to prevent the contamination in vineyards and to define the efficacy of relevant field control strategies. On the other hand, must samples are a complex matrix; there are pulp, skins, stems and seeds called pomace or grape solids, which typically comprise between 7–23% of the total weight of the must, so that this matrix could be considered as a semi solid matrix. Immunoaffinity columns (IACs) have been widely used as a clean-up tool and their use has simplified the clean-up procedure and is highly recommended: it allows the separation of the analyte from most matrix interferences due to its specificity and analyte preconcentration, which is necessary when low limits of detection are required [33]. Different commercial IACs are available for OTA analysis. 

On the other hand, the data about OTA occurrence on Argentinean grapes and by-products are scarce. The aims of this study were: (a) to evaluate the performance of OchraStar^TM^ columns for OTA determination in musts, and (b) to evaluate OTA occurrence in musts, wines and dried vine fruit.

## 2. Materials and Methods

### 2.1. Chemicals and Reagents

Ochratoxin A standard was purchased from Sigma Aldrich (St Louis, MD, USA) and stored at −8 °C. OTA purity was >99%. Water, acetic acid, methanol and acetonitrile were HPLC grade (Merck, Darmstadt, Germany). Phosphate-buffered saline (PBS) (8.0 g NaCl, 1.44 g NaHPO_4_, 0.24 g KH_2_PO_4_, 0.2 g KCl), and polyethylene glycol (PEG) 8000 were purchased from Aldrich (Sigma-Aldrich). OchraStar^TM^ Immunoaffinity Columns (IAC) were purchased from Romer Laboratories (Union, Missouri, USA). 

### 2.2. Preparation of Standard Solutions of OTA

An OTA stock standard solution of 200 µg/mL was prepared by dissolving 0.4 mg of OTA in 2 mL of toluene: acetic acid (99:1, v/v). The concentration of the stock solution was determined by measuring the absorbance at 330 nm of a diluted solution of OTA in benzene: acetic acid (99:1, v/v). The solutions were used to calibrate the fluorescence detector response. Working standard solutions from 3 to 10 ng/mL of OTA were prepared by evaporation of known volumes of the stock solution under N_2_ stream, followed by dissolution in methanol HPLC grade [29]

### 2.3. Column Capacity

Different amounts of OTA, from 2 to 200 ng, were added to the immunoaffinity column by loading 10 mL of a solution containing 5% NaHCO3 and 1% PEG 8000. The capacity of the OchraStar^TM^ columns was determined by comparing (duplicate measurements) the amount of OTA added to the immunoaffinity column with the amount bound [34]. 

### 2.4. Extraction and Clean-up with IAC

In order to evaluate the performance of the OchraStar^TM^ columns for OTA extraction and clean-up from the must samples, different procedures were evaluated for toxin extraction previous to the clean-up of the samples using the OchraStar^TM^ columns (IAC), as follows:

(a) Extraction with a solution containing acetonitrile: water (60:40) and clean up with IAC. Briefly, 25 g of must was diluted with 100 mL of acetonitrile: water (60:40), mixed, and filtered to remove particulate matter. An 8 mL portion of this dilution was evaporated to dryness and resuspended in 56 mL of PBS (acetonitrile never exceeds 7.5% v/v); finally the pH value was adjusted to 7.4 and added to the IAC. The IAC was washed twice with 10 mL of ammonium acetate (AcNH_4_) 0.2 M. Ochratoxin A was eluted from the column with 1.5 mL of methanol (HPLC grade), at a flow rate of 1–2 drops per second.
(b) Must dilution with PBS and clean up with IAC. Briefly, 8 g of must was diluted with 56 mL of PBS buffer, mixed, filtered to remove particulate matter, and added to the IAC. The column was washed twice with 10 mL of ammonium acetate (AcNH_4_) 0.2 M. OTA was eluted from the column with 1.5 mL of methanol: acetic acid (98:2), at a flow rate of 1–2 drops per second.
(c) Must dilution with water solution containing polyethylene glycol 8000 (1%) and NaHCO_3_ (5%) and clean-up on IAC according to the methodology proposed by Visconti *et al.* (1999) [34] for wine samples. In brief, must was diluted with water solution containing 1% PEG and 5% NaHCO_3_, mixed, filtered to remove particulate matter, and a 10 mL portion was taken and added to the IAC. The IAC was washed with 5 mL of an aqueous solution of 2.5% NaCl and 0.5% NaHCO_3_ and then with 5 mL double distilled water. OTA was eluted from the column with 1.5 mL of methanol (HPLC grade), at a flow rate of 1–2 drops per second.

The extracts were evaporated to dryness at 50 °C under N_2_ stream, and the residues were redissolved in 250 µL of the HPLC mobile phase.

Ochratoxin A extraction from red wine samples was done using the official method proposed by the AOAC [29]. Briefly, 10 mL of wine sample was diluted with 10 mL of an aqueous solution containing 5% NaHCO3 and 1% PEG 8000. This procedure is not an extraction ‘sensu stricto’ because many components of the sample matrix are present before clean-up. The pH was adjusted to 7.4 with 1 M solution of NaOH. The resulting solution was filtered through a Whatman glass microfiber filter to remove any solid present. A 10 mL portion was taken and added to the IAC. The column was washed with 5 mL of an aqueous solution containing 2.5% NaCl and 0.5% NaHCO_3_ and then with 5 mL double distilled water. OTA was eluted from the column with 1.5 mL of methanol (HPLC grade), again at a flow rate of 1–2 drops per second. The extracts were evaporated to dryness at 50 °C under N_2_ stream and the residues were redissolved in 250 µL of the HPLC mobile phase.

OTA extraction from dried vine fruits was done using the methodology proposed by Möller *et al.* [35]. Briefly a 50 g portion of dried vine fruits was extracted with methanol: sodium bicarbonate at 1% (70:30, v/v) and blended at high speed for 1 min. The pouring extract was filtered to remove particulate matter, and 10 mL of extract taken and diluted with 40 mL of PBS containing 0.01% Tween 20. The diluted extract was filtered through a microfiber filter. A 10 mL portion was taken and added to an IAC. The column was washed with 10 mL PBS containing 1% Tween 20 and then with 10 mL double distilled water. OTA was eluted from the column with 1.5 mL of methanol (HPLC grade), at a flow rate of 1–2 drops per second (according to manufacturer’s recommendations). The extracts were evaporated to dryness at 50 °C under N_2_ stream and the residues were solved in 250 µL of the HPLC mobile phase.

### 2.5. Ochratoxin A Detection and Quantification

The HPLC apparatus used for OTA determination was a Hewlett-Packard Series 1100 (Hewlett-Packard company, Palo Alto, CA, USA) chromatograph with a loop of 50 μL, equipped with a fluorescence detector (excitation, 330 nm; emission, 460 nm) and a Luna^TM^ C18 column (150 × 4.6 mm, 5 µm particle size; Phenomenex ®, connected to a guard column Security Guard^TM^ filled with the same phase (20 × 4.6 mm, 5 µm particle size; Phenomenex^TM^). The mobile phase acetonitrile:water:acetic acid (99:99:2, v/v/v) was pumped at 1.0 mL/min. OTA was quantified on the basis of HPLC fluorometric response compared with OTA standard using a data module Hewlett Packard Kayak XA (HP ChemStation Rev. A.06.01). 

### 2.6. Assay: Spiking and Recovery of OTA in Musts, Wines and Dried Vine Fruits

OTA-free samples (50 g of dried vine fruits, 20 mL of wines and 20 g of must) contained in a 250 mL Erlenmeyer flask were spiked with standard solutions of OTA, an equivalent of 5, 10 and 20 ng OTA g^−1^ for dried vine fruits and to 2, 5 and 10 ng OTA mL^−1^ or g^−1^ for wines and musts, respectively. Spiking was carried out in triplicate and a triplicate analysis of the blank sample was carried out. After leaving the samples for 16 h, extraction solvent was added and the OTA concentration was determined using the protocols previously described. Recovery percentage was calculated for each substrate.

### 2.7. Origin of Samples for OTA Natural Occurrence Determination

Must samples (32) were obtained from two regions: Mendoza, which represents the most important wine producing region in Argentina where several oases where grapes are cultivated [36], and Chubut, which represents a new grape-growing region. Samples (500 mL) were taken in sterile flasks directly from the winery during tank filling. The must samples were frozen at −20 °C until analysis.

Red wine samples (47) were obtained during the 2008 vintage. The sampling areas were: North of Mendoza province (Lavalle), North-West of Mendoza province (Uco Valley), High zone of Mendoza River (ZARM), South Mendoza (San Rafael), San Juan province (Tulum Valley), La Rioja Province (Famatina Valley-Chilecito) and Neuquén-Río Negro provinces. Wine samples (750 mL) were obtained from the cellar during bottling and stored at 4 °C until analysis.

Fifteen samples of dried vine fruits were collected from San Juan province, the main dried vine fruit producer region. All the dried vine fruits samples were muscatel variety without seed grapes and respected export quality criteria. 

## 3. Results and Discussion

**Figure 1:**
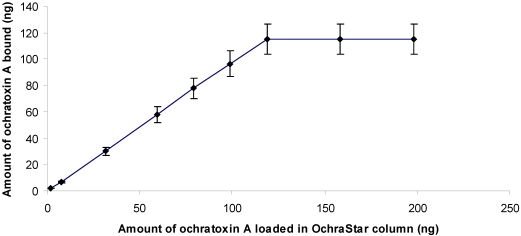
Binding performance of anti-ochratoxin A antibodies used in the OchraStar^TM^ immunoaffinity columns. Averages of duplicate measurements (± SD) are represented.

OchraStar^TM^ columns showed a good performance for cleaning up all the substrates analyzed in this study for OTA contamination. The column capacity was found to be about 100 ng of OTA. Above this level no increase of the fluorescence response was observed, indicating saturation of anti-ochratoxin A binding sites of the monoclonal antibodies. Considering the column capacity, the range of applicability of the methods for musts, red wines and dried vine fruits ranged from 2 ng/g to 100 ng/g (Figure 1).

For must samples, three extraction procedures were evaluated prior to the clean-up step. The mean toxin recovery using extraction with acetonitrile: water and clean-up with OchraStar^TM^ columns (procedure a) was 98.9% in the spiking range. The precision expressed as relative standard deviation within the laboratory (RSDr) was 4.8%. On the other hand, when the must samples were diluted with PBS and cleaned up with OchraStar^TM^ columns (procedure b), the mean recovery was lower than (a) procedure (40.35%), while the RSDr was estimated to be 22.3%. Finally, must dilution with PEG/NaHCO_3_ and clean up with OchraStar^TM^ columns using the procedure (c) showed that the mean recovery was 100.3% and the precision mean value (RSDr) was 2.35% (Table 1). 

The results using OchraStar™ columns for clean-up (with the exception of procedure b) satisfied the criteria established by IUPAC/AOAC/ISO/CEN standards. To recognize an analytical method as an official standard for the purpose of enforcement, the recovery and the RSDr values needed to be within a range of 70 to 110% and <20% respectively [37]. The recovery values obtained were homogeneous throughout the entire range of the spiking OTA concentrations assayed. The quantification limit (LOQ) based on a signal: noise 10:1, the recovery and the RSDr were acceptable (0.1 ng/mL, ≥90% and ≤10%, respectively) (Table 1). Since the procedure (c) requires no solvent extraction and showed a good performance, it was selected to evaluate the natural occurrence of OTA in the must samples.

In the particular case of red wine samples, the mean recovery was 90 ± 2% while the precision (RSDr) was 2.5%, and the detection limit was 0.01 ng/mL (Table 1)

The mean recovery of the method used for dried vine fruits was 95 ± 2.5% and the precison (RSDr) was 2.6%. The lowest limit of detection was 0.05 ng/g (Table 1). 

For these substrates the recoveries and precision obtained were similar to the previously obtained by the official methodology proposed by the AOAC. 

The analysis of natural occurrence of OTA in 32 must samples showed that OTA was present only in three samples (9%) in low levels (Table 2). The low number of contaminated must samples and the low OTA levels detected could be related to the low incidence of the principal ochratoxigenic species *A. carbonarius* found in grapes, and to non-conductive climatic conditions during the harvest season 2008/2009 [38]. Our results agree with those presented by El Khoury *et al.* [39,40], who studied the presence of OTA on must samples from Lebanon during the 2004 and 2005 vintages. They found low levels of OTA during the 2004 vintage and no contamination in 2005. They also correlated their results with low incidence of *A. carbonarius* on the vineyards evaluated. There are previous reports on the contamination of must samples with OTA, but they were obtained from artificially contaminated grapes [24,41,42]. This makes comparison with our results difficult since our must samples were obtained from grapes without artificial contamination. 

**Table 1:** Mean recovery of OTA from grapes by product samples fortified with three toxin levels (*n* = 3).

Grapes by Product	Extraction/Clean Up Procedure	Mean Recovery (%) ± SD	RSDr (%)	LOD	LOQ
Must	(a) Extraction with Acetonitrile:Water (60:40) + OchraStar^TM^ clean-up.	98.9 ± 4.84	4.80	0.05 ^a^	0.1 ^a^
	(b) Must diluted with PBS + OchraStar^TM^ clean-up.	40.35 ± 12.42	22.3	0.05 ^a^	0.1 ^a^
	(c) Dilution with PEG-NaHCO_3_ + OchraStar^TM^ clean-up	100.3 ± 2.4	2.35	0.05 ^a^	0.1 ^a^
Wine	Official method proposed by Visconti *et al.* 2001 [29]	90 ± 2	2.50	0.01 ^a^	0.1 ^a^
Dried vine fruits	Method proposed by Möller *et al.* 2003 [35]	95 ± 2.5	2.60	0.1 ^b^	1 ^b^

^a^ ng/mL; ^b^ ng/g.

**Table 2:** Occurrence and ochratoxin A levels in must samples from Argentina.

Region of Origin	Number of Positive Samples/Total samples	Range of OTA (ng/mL)	Mean OTA Levels (ng/mL)
Mendoza province	3/30	≤0.1–0.16	0.12 ± 0.04
Chubut province	0/2	ND	ND

ND: Not Detected (≤LOD); LOD: 0.05 ng/mL; LOQ: 0.1 ng/mL.

In terms of OTA content in red wine samples, 8.5% of the samples were positive, and the levels detected ranged from 0.02 to 4.82 ng/mL, though only two samples showed levels higher than 2 ng/mL (Table 3). From samples collected in La Rioja province, only one was OTA positive (17%). Mendoza province was divided into four regions according to the climatic conditions data, as follows: Higher Zone of Mendoza River (ZARM), Uco-Valley, North of Mendoza and South of Mendoza. Only in 9% of the samples from the Uco Valley were positive for OTA and the levels detected were lower than 2ng/mL. Samples from North and South of Mendoza were OTA contaminated in percentages of 17% and 14%, respectively, showing levels higher than 2 ng/mL. Samples from San Juan and Río Negro provinces showed no OTA contamination (Table 3). 

The percentage of samples contaminated with OTA—from highest to lowest percentage—were La Rioja > North Mendoza > South Mendoza > Mendoza Uco Valley > Mendoza ZARM = San Juan = Neuquén-Río Negro. The differences in OTA contamination in the samples analyzed in this study could be explained by considering the climatic conditions on the vineyards along the sampling areas. Although all the viticultural areas have arid-desert climates, the northern regions, such as La Rioja and North of Mendoza, are warmer and drier than the southern ones, with mean temperatures of 30 °C and a mean relative humidity of 56% during the grape maturation period. Mendoza ZARM, San Juan and Neuquén-Río Negro have a high thermal amplitude and mean temperatures between 20–25 °C and a mean relative humidity between 52–61% [38,43]. Previous studies have demonstrated that the meteorological conditions and geographic area can contribute to variation in grape fungal colonization and OTA contamination [38,43,44,45,46,47]. It is interesting to remark that the percentage of OTA positive samples in musts and wines from Mendoza province were similar (9%). 

Furthermore, OTA was detected in wine for the first time in 1996 [18]. Later surveys conducted in Europe showed that it was a problem mainly for Southern Europe, where climatic conditions favor the growth of OTA-producing fungi in grapes. The incidence and levels of contamination in this region was higher than that in wines produced in Northern and Central Italy [19]. A high incidence of contamination (from 40% to 87%) was reported in all surveys—with the exception of the Australian one that show an incidence of 15% [49]. The maximum OTA level was recorded in Italy (15.6 µg/kg) [19]. In our study there was a lower incidence of OTA contaminated samples (9%), with a maximum level of 4.82 ng/mL found.

**Table 3:** Ochratoxin A levels in wines from different growing regions of Argentina.

Region of Origin	Positive Sample/Total Samples	Concentration Range of OTA (ng/mL)
Famatina Valley (La Rioja province)	1/6	2
Tulum Valley (San Juan province)	0/6	ND
ZARM (Mendoza Province)	0/8	ND
Uco Valley (Mendoza province)	1/11	0.02
North of Mendoza	1/6	3.14
South of Mendoza	1/7	4.82
Neuquén – Río Negro	0/3	ND
Total	4/47	0.02–4.82

ND: Not Detected (≤LOD); LOD: 0.01 ng/mL; LOQ: 0.1 ng/mL.

Of the dried vine fruit samples, 9 of 15 (60%) were contaminated with OTA, in levels ranging between 0.26–20.28 ng/g (Table 4). In Argentina, similar results were obtained by Magnoli *et al.* [6], which showed that OTA was present in about 74% of the dried vine fruit samples. The highest OTA concentration was of 14 ng/g in a black dried fruit sample (ranging from 1.5 to 14 ng/g).

OTA has also been reported to be naturally occurring in dried vine fruits in other countries. MacDonald *et al.* [48] showed a high incidence of OTA (88%) in sultanas, raisins and currants imported to the UK, with a maximum level of 53.6 µg/kg. Stefanaki *et al.* [51] also showed OTA presence in 81 samples of Greek dried vine fruits. OTA was found in 79% of the current samples and 62% of the sultanas samples examined, showed average levels of 2.8 and a maximum of 13.8 ng/g and 2.1 and a maximum of 13.2 ng/g, respectively. It is evident that dried vine fruit (currants, raisins and sultanas) can be highly contaminated with OTA. This substrate can be an important dietary source of OTA for people with high levels of consumption, in particular children. In fact, dried vine fruits are commonly included into breakfast cereals and other food items. This could thus represent an important route of entry into the human food chain.

**Table 4:** Occurrence of ochratoxin A in black dried vine fruits from San Juan Province, Argentina.

Number of Positive Samples/ Total Samples	Rangeof OTA (ng/g)	Mean OTA Levels (ng/g)
2/16	8.66–20.28	14.47 ± 8.21
8/16	0.26–3.1	1.07 ± 1.01
6/16	ND	ND

ND: Not Detected (≤LOD); LOD: 0.1 ng/g; LOQ: 1 ng/g.

*In vitro* studies on the effects of temperature on growth rates and OTA production by *Aspergillus* section *Nigri* species have shown that temperatures ranging from 30 to 37 °C are optimum for both parameters [19,52,53,54,55,56]. This increases the OTA contamination risk in grapes and wines in areas with those temperatures. This effect on OTA risk contamination related to the temperature of the region has been found by Chiotta *et al.* [38] on the natural occurrence of OTA in grapes in different regions of Argentina. 

Although the incidence of OTA in grapes and by-products depends on the type of substrate, the availability of reliable analytical methods for this toxin determination in must, wine and relevant grapes by-products is important for the risk management of OTA contamination in the food chain. To take prompt corrective action, the availability of rapid methods is necessary in wineries for screening the whole production.

## 4. Conclusions

The results obtained in the present study showed that the immunoaffinity columns (OchraStar^TM^) perform well for the clean-up of red musts, red wines and dried vine fruits. In addition, the analysis of the natural occurrence of OTA in the red must and the red wine samples showed a low percentage of contaminated samples with low mean levels of OTA (0.12 and 0.37 ng/mL, respectively) while 60% of the dried vine fruit samples were contaminated with OTA, with levels from 0.26 to 20.28 ng/g.
